# Celastrol attenuates ox-LDL-induced mesangial cell proliferation via suppressing NLRP3 inflammasome activation

**DOI:** 10.1038/s41420-019-0196-0

**Published:** 2019-07-05

**Authors:** Zhenzhen Sun, Yuanyuan Li, Yun Qian, Mengying Wu, Songming Huang, Aihua Zhang, Yue Zhang, Zhanjun Jia

**Affiliations:** 1 Department of Nephrology, Children’s Hospital of Nanjing Medical University, Guangzhou Road #72, 210008 Nanjing, China; 2 Jiangsu Key Laboratory of Pediatrics, Nanjing Medical University, 210029 Nanjing, China; 3 Nanjing Key Laboratory of Pediatrics, Children’s Hospital of Nanjing Medical University, 210008 Nanjing, China

**Keywords:** Kidney diseases, Cell biology

## Abstract

Mesangial cell (MC) proliferation is one of the important pathological features of obesity-associated nephropathy with unknown etiology. Excessive MC proliferation can cause glomerulosclerosis and renal function loss. Thus, targeting MC proliferation may be a potential strategy for the treatment of obesity-associated kidney disease. The present study was undertaken to investigate the role of celastrol in MC proliferation induced by ox-LDL, as well as the potential mechanisms. Following ox-LDL treatment, MC proliferation was induced and the NLRP3 inflammasome was activated, as evidenced by increased NLRP3 levels, caspase 1 activity, and IL-18 and IL-1β release. Significantly, NLRP3 siRNAs inhibited MC proliferation and delayed cell cycle progression, as indicated by the cell cycle assay and the expression of cyclin A2 and cyclin D1. Given the anti-inflammatory effect of celastrol, we pretreated MCs with celastrol before ox-LDL treatment. As expected, celastrol pretreatment strikingly inhibited NLRP3 inflammasome activation and MC proliferation triggered by ox-LDL. In summary, celastrol potently blocked ox-LDL-induced MC proliferation, possibly by inhibiting NLRP3 inflammasome activation. These findings also suggest that celastrol may be a potential drug for treating proliferative glomerular diseases related to obesity and lipid disorders.

## Introduction

Considerable evidence has shown that obesity-related metabolic syndrome (MetS) is a major risk factor for glomerulopathy^1–6^. Lipid metabolism disorders are commonly associated with hyperlipidemia and the glomerular accumulation of atherogenic lipoproteins, of which the oxidatively modified low-density lipoprotein (ox-LDL) is a compelling contributor to glomerular mesangial proliferation, inflammation and extracellular matrix (ECM) expansion, which ultimately leads to glomerulosclerosis and nephron loss^7–12^. Thus, targeting mesangial cell (MC) proliferation in obesity and MetS is of importance for treating or preventing proliferative glomerulopathy.

The nucleotide binding and oligomerization domain-like receptor family pyrin domain containing three (NLRP3) inflammasome was recently reported to be involved in various diseases, and the activation of the NLRP3 inflammasome results in the cleavage of pro-IL-1β and pro-IL-18 into the pro-inflammatory cytokines IL-1β and IL-18^13–15^. In kidney MCs, recent research has demonstrated that activation of the NLRP3 inflammasome contributes to high-glucose-induced rat mesangial cell inflammation and immunoglomerulonephritis^11,16,17^. However, the role of the NLRP3 inflammasome in lipid-related mesangial cell proliferation remains unclear.

Celastrol is a triterpene extracted from the traditional Chinese medicinal plant Tripterygium wilfordii Hook F and is listed by the journal Cell as one of the five traditional medicinal compounds most likely to be developed as a modern drug^18–20^. Accumulating evidence indicates that celastrol exhibits potent anti-inflammation, anti-immunity, and anti-cancer effects^18,21–23^. Previous studies have found that celastrol can inhibit the activation of the NLRP3 inflammasome in the innate immune defense^18,24,25^. Additionally, celastrol-albumin nanoparticles are effective in treating mesangioproliferative glomerulonephritis in rats stimulated by anti-Thy1.1^26^. However, the mechanism underlying the effect of celastrol against mesangial cell overproliferation in such models is not understood. Moreover, whether celastrol has a similar effect against lipid-induced MC proliferation still needs to be studied. Considering the contribution of the NLRP3 inflammasome to MC pathology and the effect of celastrol on NLRP3 inflammasome activation, we hypothesize that celastrol may be beneficial against lipid disorder-associated MC proliferation and NLRP3 inflammasome activation.

## Results

### Ox-LDL-induced MC proliferation

**Fig. 1:**
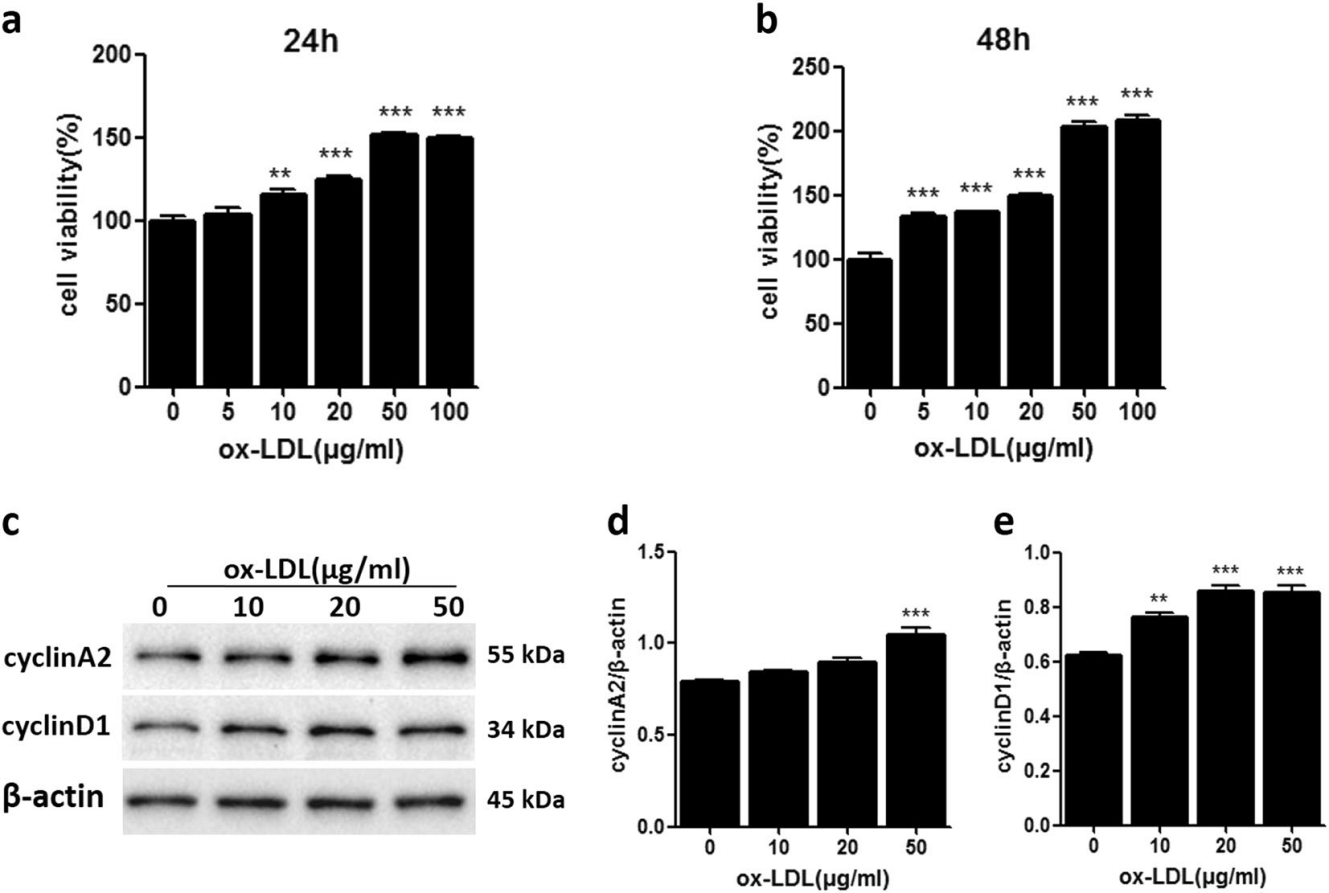
Ox-LDL-induced MC proliferation. **a**, **b** CCK-8 assay for the proliferative activity of MCs treated with various concentrations of ox-LDL for 24 h or 48 h. **c** Western blot analysis of cyclin A2 and cyclin D1. **d**, **e** Densitometric analysis of the western blots. The data are shown as the mean ± SD of three replicates. **P < 0.01 vs. the “0” control group, ***P < 0.001 vs. the “0” control group

To confirm that ox-LDL treatment can induce MC proliferation, in the present study, we treated MCs with various concentrations of ox-LDL (0, 5, 10, 20, 50, and 100 μg/ml) for 24 or 48 h. As expected, ox-LDL-induced MC proliferation in a dose- and time-dependent manner (Fig. 1a, b). We also measured the expression of cyclin A2 and cyclin D1, which belong to the cyclin family and are involved in regulating cell cycle progression, after ox-LDL stimulation. Western blot results showed that ox-LDL significantly increased cyclin A2 and cyclin D1 protein expression, especially at 50 μg/ml (Fig. 1c–e), suggesting that the mechanism by which ox-LDL induces MC proliferation may involve the cell cycle.

### Celastrol attenuated MC proliferation induced by ox-LDL

**Fig. 2:**
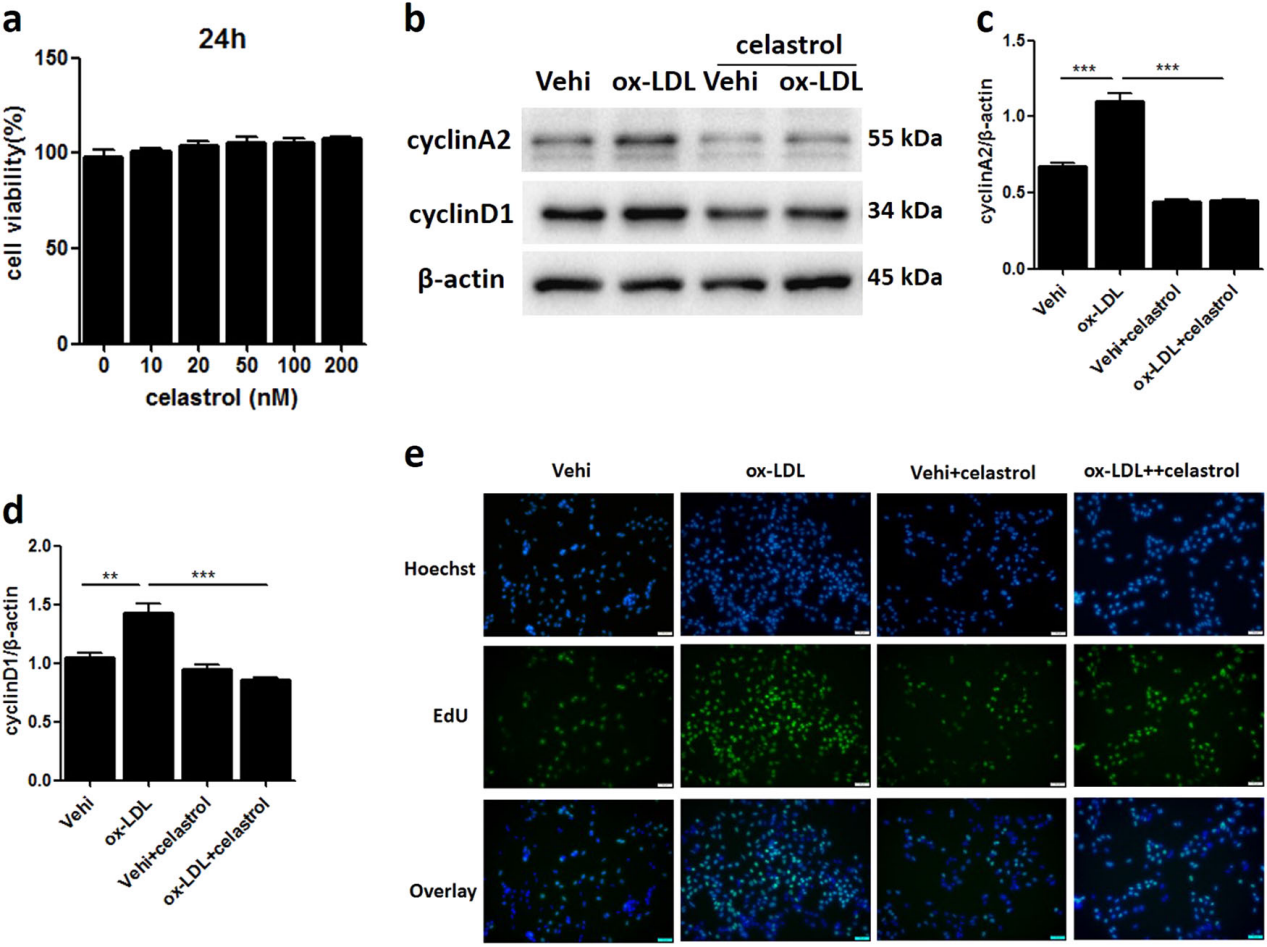
Celastrol attenuated MC proliferation induced by ox-LDL. **a** CCK-8 assay to determine the viability of MCs treated with various concentrations of celastrol for 24 h. **b** Protein expression of cyclin A2 and cyclin D1 was measured by western blot. **c**, **d** Densitometric analysis of the western blots. **e** MC proliferative activity was measured by the EDU test. Scale bars, 100 μM. Vehi, vehicle. The data are shown as the mean ± SD of three replicates. **P < 0.01, ***P < 0.001

We investigated whether celastrol is effective in preventing ox-LDL-induced MC proliferation. First, we tested a series of concentrations of celastrol to determine its influence on MC viability. Celastrol did not have an obvious cytotoxic effect on MCs, even at 100 mM (Fig. 2a). Then, 50 mM celastrol was chosen for subsequent experiments. Following celastrol treatment, the ox-LDL-induced upregulation of cyclin A2 and cyclin D1 protein expression was significantly blocked (Fig. 2b–d). By the EDU test, we observed that celastrol decreased cell proliferation induced by ox-LDL (Fig. 2e).

### Celastrol blocked ox-LDL-induced cell cycle progression in MCs

**Fig. 3:**
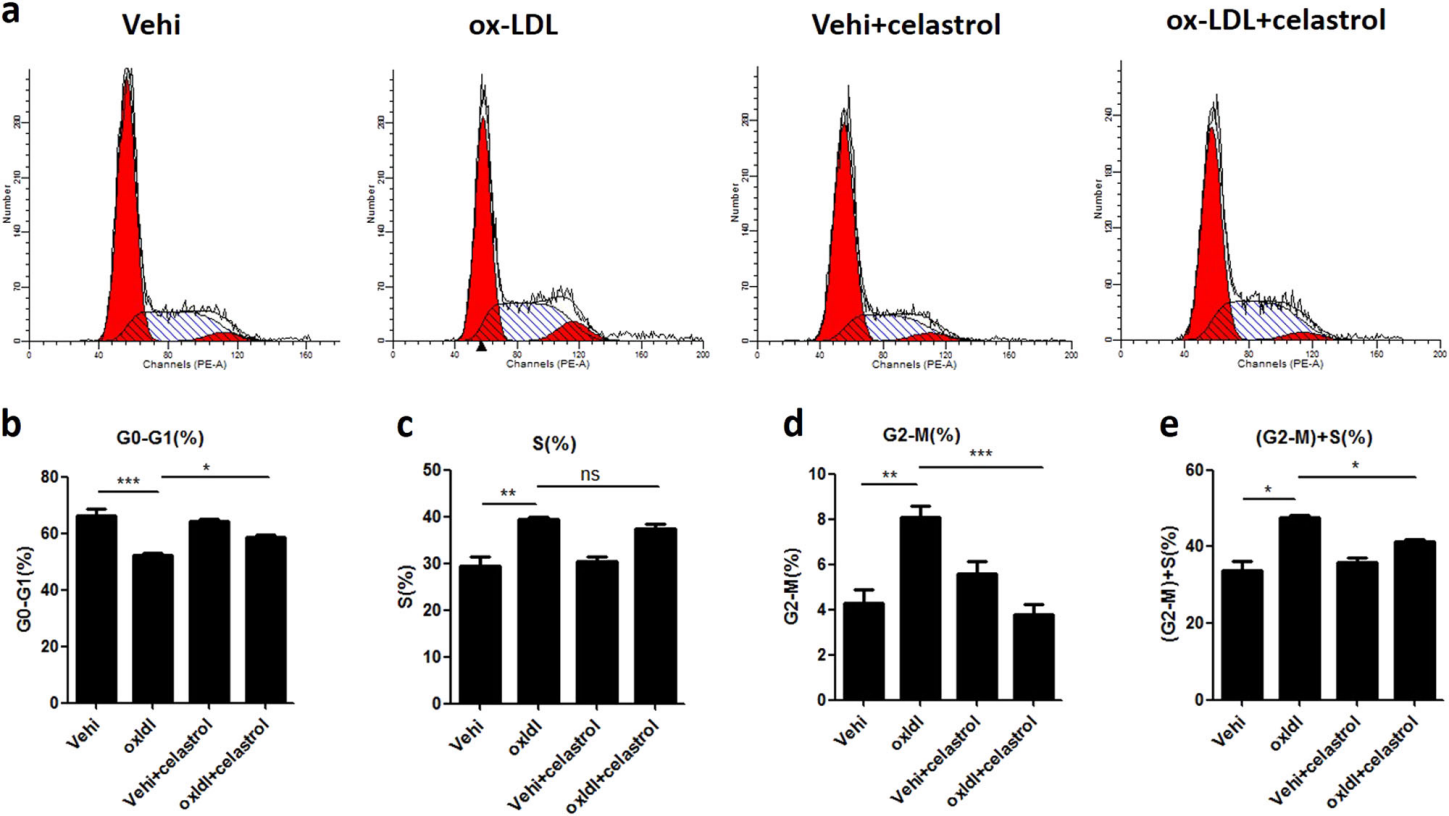
Celastrol blocked ox-LDL-induced cell cycle progression in MCs. **a** Representative images of the stages of the cell cycle, which were detected by flow cytometry. **b**–**e** Percentage of cells in the different cell cycle phases. The data are shown as the mean ± SD of three replicates. ns, not significant. *P < 0.05, **P < 0.01, ***P < 0.001

Owing to the known role of cell cycle progression in cellular proliferation and the above evidence showing the effect of celastrol on the regulation of cyclin A2 and cyclin D1, we further explored the action of celastrol on cell cycle progression. The number of cells in each cell cycle phase was analyzed by DNA propidium staining. As shown in Fig. 3, ox-LDL increased the number of cells both in the S phase and G2-M phase and decreased the number of cells in the G0-G1 phase, indicating that it promoted cell cycle progression. Strikingly, celastrol decreased the number of cells in the G2-M phase, and there was a trend towards a decreased number of cells in the S phase (Fig. 3a–e). These results strongly suggest that celastrol is a potent blocker of cell cycle progression in MCs during challenge with ox-LDL.

### Ox-LDL triggered NLRP3 inflammasome activation in MCs

**Fig. 4:**
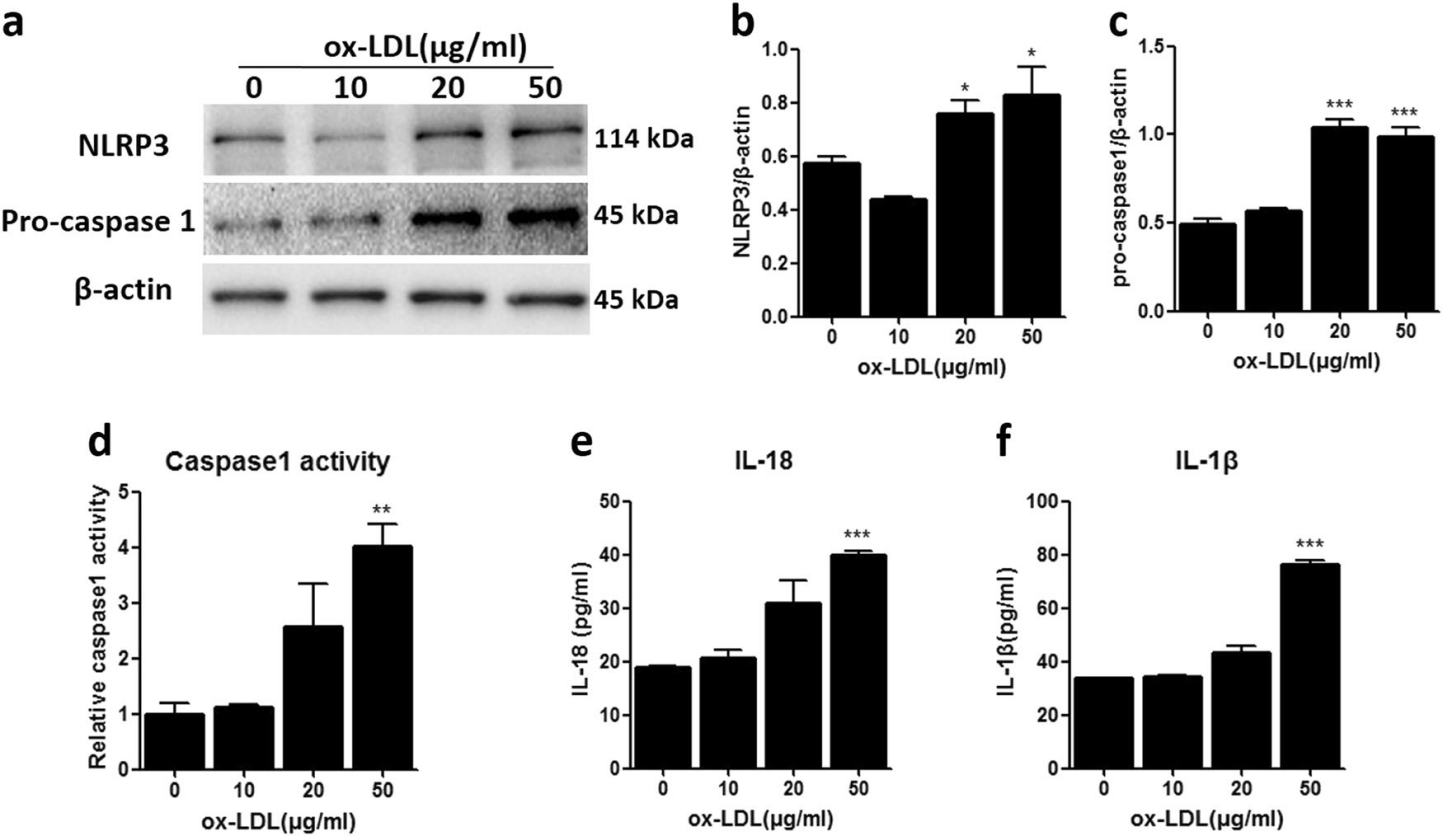
Ox-LDL triggered NLRP3 inflammasome activation in MCs. **a** Protein expression of NLRP3 and pro-caspase 1 was measured by western blot. **b**, **c** Densitometric analysis of the western blots. **d** Caspase 1 activity was measured by a caspase 1/ICE colorimetric assay. **e**, **f** The levels of IL-18 and IL-1β in the MC culture medium were detected by ELISA. The data are shown as the mean ± SD of three replicates. *P < 0.05, **P < 0.01, ***P < 0.001 vs. the “0” control group

A previous study suggested the contribution of the NLRP3 inflammasome to high-glucose-induced MC proliferation. Therefore, we detected the effect of ox-LDL on the activation of the NLRP3 inflammasome. As expected, ox-LDL upregulated the protein expression of NLRP3 and pro-caspase 1 (Fig. 4a–c), enhanced caspase 1 activity (Fig. 4d) and increased the secretion of IL-18 and IL-1β in the MC culture medium (Fig. 4e, f). These results demonstrate that ox-LDL can activate the NLRP3 inflammasome in MCs.

### Celastrol inhibited NLRP3 inflammasome activation triggered by ox-LDL in MCs

**Fig. 5:**
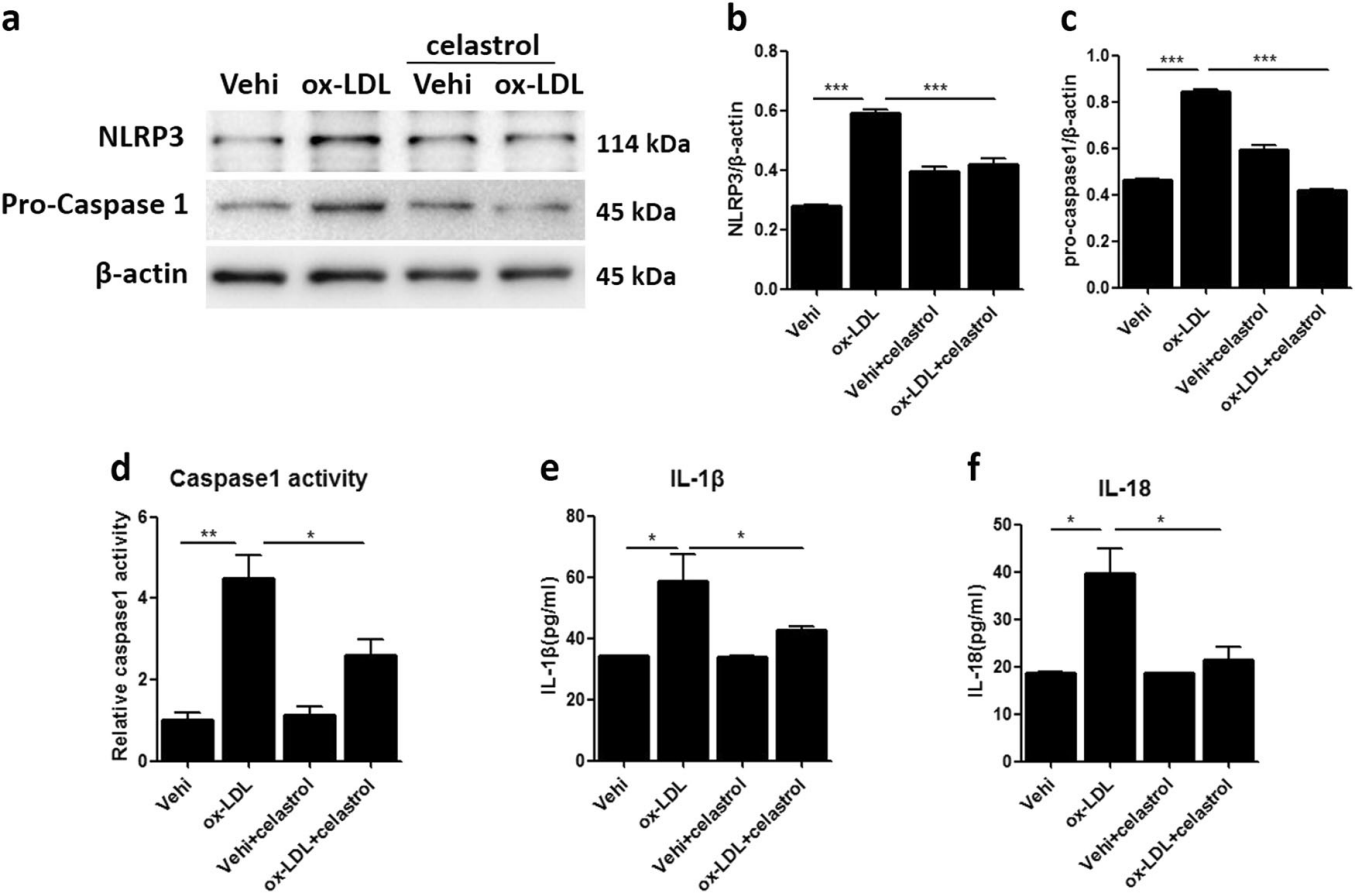
Celastrol inhibited NLRP3 inflammasome activation triggered by ox-LDL in MCs. **a** Representative western blot images of the protein expression of NLRP3 and pro-caspase 1. **b**, **c** Densitometric analysis of the western blots. **d** Relative caspase 1 activity was determined by a caspase 1/ICE colorimetric assay. **e**, **f** ELISA for the release of IL-18 and IL-1β in the MC culture medium. The data are shown as the mean ± SD of three replicates. *P < 0.05, **P < 0.01, ***P < 0.001

To evaluate the role of celastrol on NLRP3 inflammasome activation, MCs were treated with celastrol before ox-LDL stimulation. As shown in Fig. 5a–c, celastrol pretreatment suppressed the protein expression of NLRP3 and pro-caspase 1 induced by ox-LDL. Furthermore, caspase 1 activity was also significantly inhibited by celastrol (Fig. 5d). Consistent with these results, the release of IL-18 and IL-1β in the MC culture medium was reduced significantly (Fig. 5e, f). The above results demonstrate a strong action of celastrol on inhibiting NLRP3 inflammasome activation in MCs triggered by ox-LDL.

### Silencing NLRP3 inhibited NLRP3 inflammasome activation triggered by ox-LDL in MCs

**Fig. 6:**
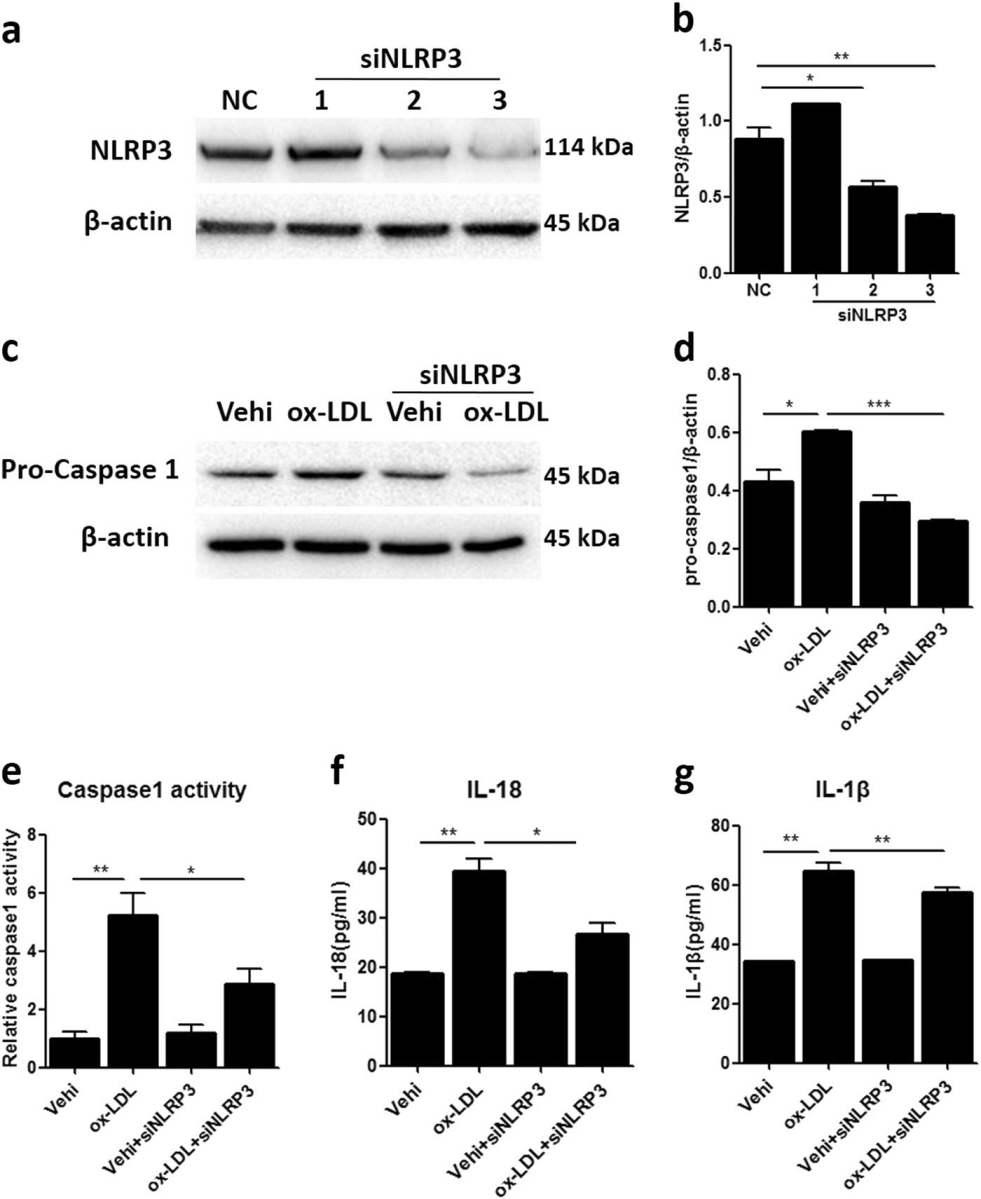
Silencing NLRP3 inhibited NLRP3 inflammasome activation triggered by ox-LDL in MCs. **a**–**c** Western blot analysis for the protein expression of NLRP3 and pro-caspase 1. NC, negative control. **b**–**d** Quantitative analysis of the western blots. **e** Caspase 1 activity was measured by a caspase 1/ICE colorimetric assay. **f**, **g** The levels of IL-18 and IL-1β in the MC culture medium were detected by ELISA. The data are shown as the mean ± SD of three replicates. *P < 0.05, **P < 0.01, ***P < 0.001

To further explore the role of the NLRP3 inflammasome in MC proliferation induced by ox-LDL, NLRP3 small-interfering RNA (siRNA) was used in MCs. As shown by the data, two NLRP3 siRNA candidates markedly blocked NLRP3 protein expression (Fig. 6a, b). Then, we transfected MCs with a mixture of the two effective NLRP3 siRNAs before ox-LDL treatment and found that NLRP3 deficiency blocked the upregulation of pro-caspase 1 induced by ox-LDL (Fig. 6c, d). Furthermore, enhanced caspase 1 activity and the release of IL-18 and IL-1β induced by ox-LDL in MCs were significantly blunted by celastrol treatment (Fig. 6e–g). These data indicate that silencing NLRP3 can effectively block the activation of the NLRP3 inflammasome triggered by ox-LDL in MCs.

### Inactivation of the NLRP3 inflammasome attenuated MC proliferation induced by ox-LDL

**Fig. 7:**
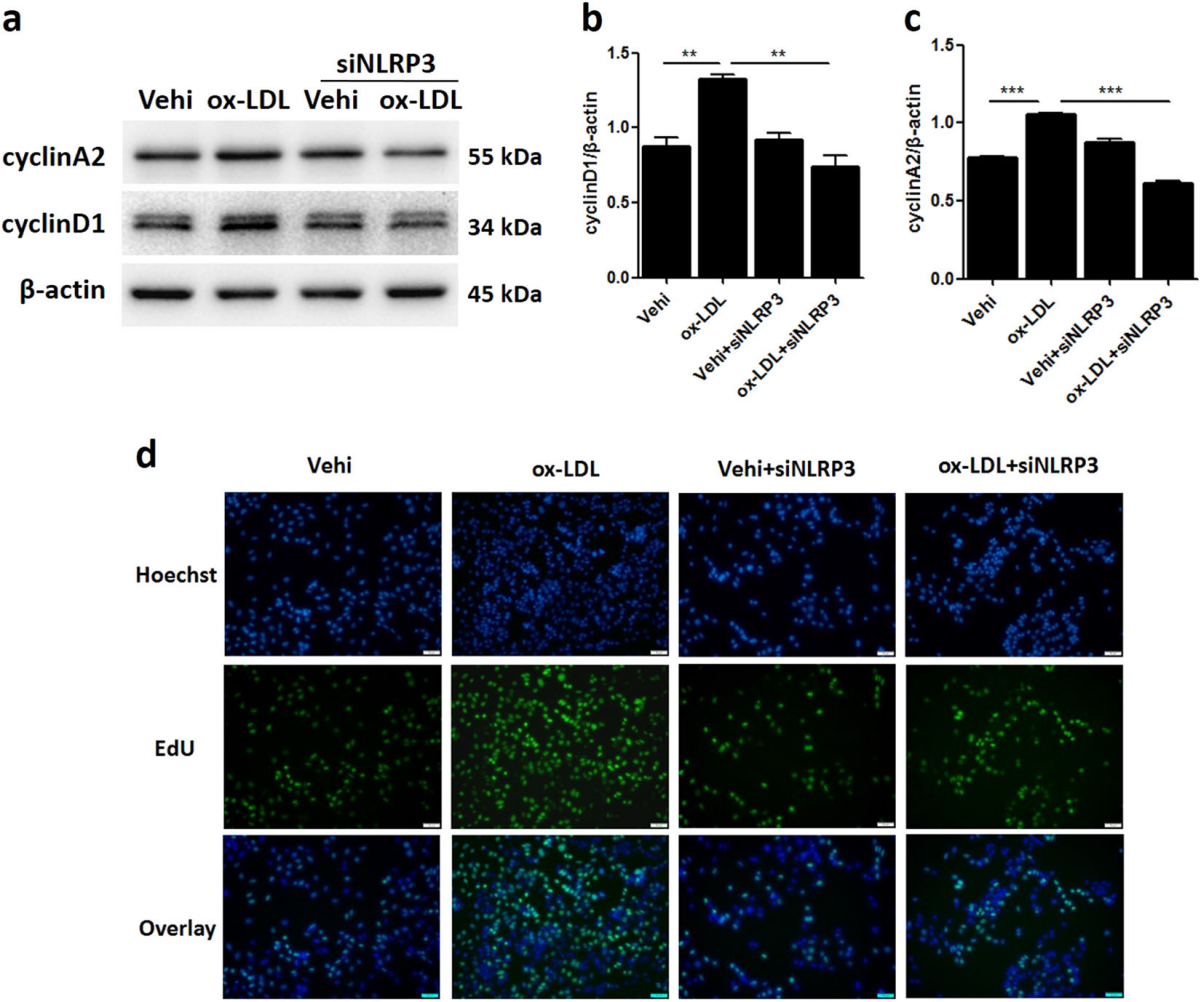
Inactivation of the NLRP3 inflammasome attenuated MC proliferation induced by ox-LDL. **a** Western blot images of cyclin A2 and cyclin D1 expression. **b**, **c** Quantitative of the western blots. **d** EDU assay images visually showing cell numbers and proliferative activity. Scale bars, 100 μM. The data are shown as the mean ± SD of three replicates. **P < 0.01, ***P < 0.001

Next, we examined the effect of NLRP3 inflammasome in activation on MC proliferation induced by ox-LDL. As expected, inactivating the NLRP3 inflammasome by silencing NLRP3 significantly blocked the upregulation of cyclin A2 and cyclin D1 protein expression caused by ox-LDL (Fig. 7a–c). By the EDU test, we observed that NLRP3 inflammasome inactivation obviously suppressed the proliferative activity of MCs induced by ox-LDL (Fig. 7d). These data suggest a pathogenic role for the NLRP3 inflammasome in ox-LDL-induced MC proliferation.

### Inactivation of the NLRP3 inflammasome blocked ox-LDL-induced cell cycle progression in MCs

**Fig. 8:**
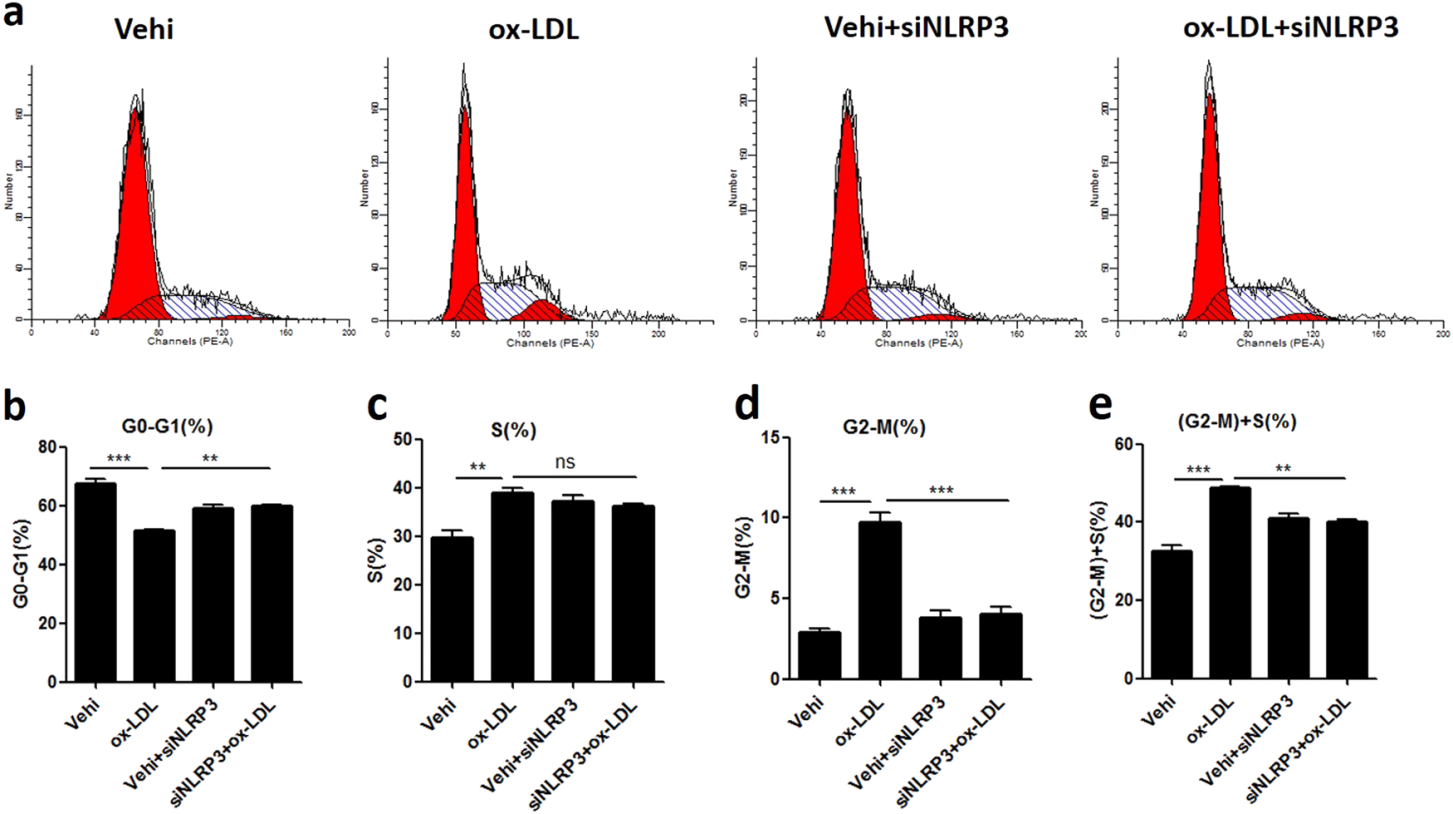
Inactivation of the NLRP3 inflammasome blocked ox-LDL-induced cell cycle progression in MCs. **a** Representative images of the states of the cell cycle, as detected by flow cytometry. **b**–**e** Percentage of cells in the different cell cycle phases. The data are shown as the mean ± SD of three replicates. ns, not significant; **P < 0.01, ***P < 0.001

Finally, we determined the effect of NLRP3 inflammasome inactivation on cell cycle progression induced by ox-LDL in MCs. As shown in Fig. 8, during challenge with ox-LDL, the number of cells in the G2-M phase was decreased significantly in the MCs transfected with NLRP3 siRNAs, and there was a trend towards a decreased number of cells in the S phase (Fig. 8a–e). The above results indicates that NLRP3 inflammasome activation contributes to ox-LDL-induced cell cycle progression in MCs.

## Discussion

With the elevated prevalence of obesity worldwide, the occurrence of obesity-related glomerulopathy has increased. Mesangial cell proliferation is an important pathological feature of obesity-related glomerulopathy, which may contribute to glomerular hypertrophy and subsequent glomerulosclerosis and renal function decline. Therefore, finding a drug to control MC proliferation associated with obesity and hyperlipidemia is important. Here we found that ox-LDL treatment promotes MC proliferation, which is effectively attenuated by celastrol. In addition, we found that NLRP3 inflammasome activation may be involved in ox-LDL-induced MC proliferation, and the antagonizing effect of celastrol on MC proliferation may occur through the inhibition of this inflammasome.

Celastrol is a natural product with potential pharmacological activities, including anti-cancer, anti-inflammation, and anti-immunity activities. In recent years, research on celastrol has shown its effect against obesity and metabolic diseases^27–30^. Interestingly, a study showed that celastrol-albumin nanoparticles are effective in treating anti-Thy1.1-induced mesangioproliferative glomerulonephritis in rats. The study focused on determining the optimal size of celastrol-albumin nano particles to obtain more accumulation in the glomeruli, as well as their role in glomerular injury, but the detailed molecular mechanism of celastrol against mesangial cell proliferation was not defined. Additionally, whether celastrol can affect ox-LDL-induced MC proliferation has not been reported. In this study, we found that celastrol attenuates ox-LDL-induced MC proliferation and cell cycle progression, suggesting a role for celastrol in antagonizing lipid disorder-related glomerular disease.

Hyperlipidemia and the accumulation of atherogenic lipoproteins are commonly accompanied by inflammation, which is positively related to the development of glomerular mesangial proliferative diseases^3,6,9^. Additionally, previous studies have suggested that the NLRP3 inflammasome is involved in high-glucose-induced MC proliferation. However, the role of the NLRP3 inflammasome in ox-LDL-related MC proliferation is still unknown. Our data showed a notably upregulated expression of NLRP3 inflammasome components in line with increased caspase 1 activity and IL-18 and IL-1β release in MC culture medium, which confirmed that the NLRP3 inflammasome was activated in MCs stimulated with ox-LDL. NLRP3 knockdown by siRNA not only inhibited the activation of the NLRP3 inflammasome but also blocked MC proliferation induced by ox-LDL. The above results demonstrate that the NLRP3 inflammasome is an important contributor to MC proliferation induced by ox-LDL. Our data also show that celastrol inhibits NLRP3 inflammasome activation induced by ox-LDL, which suggests that celastrol inhibits ox-LDL-induced MC proliferation and cell cycle progression by blocking NLRP3 inflammasome activation.

In summary, in the present study, we report that celastrol effectively attenuates MC proliferation induced by ox-LDL, possibly through inhibiting NLRP3 inflammasome activation. The above findings strongly suggest that the clinical application of celastrol may play multiple beneficial roles in obesity and obesity-associated nephropathy.

## Materials and methods

### Reagents and antibodies

Ox-LDL was purchased from Yiyuan Biotech (Guangzhou, China). Celastrol was purchased from Sigma (St. Louis, MO). Dulbecco’s modified Eagle’s medium (DMEM), fetal bovine serum (FBS), and 0.25% trypsin-EDTA were purchased from Gibco (Invitrogen, Grand Island, NY). A cyclin A2 rabbit polyclonal antibody and a cyclin D1 mouse monoclonal antibody were purchased from Proteintech (USA). Anti-NLRP3 and anti-β-actin were provided by Abcam (Cambridge, MA). Anti-mouse caspase 1 (p20) was purchased from Adipogen (San Diego, USA).

### Cell culture

Mouse mesangial cells (MCs) were obtained from the China Center for Type Culture Collection (CCTCC, Wuhan, China) and were cultured in DMEM supplemented with 10% FBS at 37 °C with 5% CO_2_. The cells were subcultured at 80–90% confluence using trypsin-EDTA (0.25%).

### CCK-8 assay

MCs were plated in 96-well plates and treated with different doses (0, 5, 10, 20, 50, and 100 μg/ml) of ox-LDL for 24 h. Then, the cells were incubated with 10 μl of CCK-8 solution for 2 h. The optical density at 450 nm was determined.

### Western blotting

After treatment, cell lysates were collected using RIPA buffer supplemented with protease and phosphatase inhibitors. Whole protein supernatant was obtained by centrifuging at 12,000 × *g* for 15 min at 4 °C. The protein concentration was determined with a BCA protein assay kit (Beyotime, Shanghai, China). The proteins were separated by sodium dodecyl sulfate polyacrylamide gel electrophoresis and transferred onto polyvinylidene difluoride (PVDF) membranes (Bio-Rad). After blocking in defatted milk, the membranes were incubated with primary antibodies against cyclin A2, cyclin D1, NLRP3, caspase 1 (p20), and β-actin, followed by incubation with secondary antibodies. The immunoreactive bands were detected with ECL reagents using a gel imager (Bio-Rad). The signals obtained from western blotting were quantified with ImageJ software. The internal control β-actin was used to normalize loading variations.

### siRNA transfection and EDU assay

MCs were plated in 24-well plates. The cells were pretreated with celastrol (50 μM) or transfected with siNLRP3/NC, followed by treatment with ox-LDL for 24 h. At each time point, the cells were incubated with EDU solution, fixed with paraformaldehyde, and stained with Apollo 488, followed by nucleus staining using Hoechst 33342. Finally, the cells were imaged using a fluorescence microscope (Olympus, Tokyo, Japan).

### Cell cycle analysis

MCs were harvested using 0.25% trypsin, followed by 70% ethanol fixation, RNase A treatment and propidium iodide staining according to the protocol of the cell cycle detection kit (KeyGEN, Shanghai, China). The number of cells in each cell cycle phase (G0-G1, S, and G2-M) was determined by flow cytometry using a BD FACS Calibur flow cytometer (Bedford, MA), and data analysis was performed with ModiFIT 3.0 software.

### Enzyme-linked immune sorbent assay (ELISA) assay

The cell culture medium was collected after treatment and centrifuged at 2000 rpm for 10 min. The supernatant was collected for cytokine analysis. The concentrations of the cytokines IL-18 and IL-1β were determined according to the manufacturer’s instructions using ELISA kits (Elabscience, Wuhan, China).

### Caspase 1/ICE colorimetric assay

Caspase 1 activity was determined using a caspase 1/ICE colorimetric assay kit (BioVision, USA). Once the treatment was terminated, the cell lysates were collected using chilled cell lysis buffer, and the supernatant was collected after centrifugation. The protein concentration was determined with a BCA protein assay kit (Beyotime), and 100–200 μg of protein from each sample was incubated with reaction buffer and YVAD-ρNA substrate at 37 °C for 2 h. The samples were read at 405 nm using the Multiskan FC (Thermo Scientific).

### Statistical analysis

Statistical analysis was conducted using SPSS version 21.0 (Chicago, IL, USA). Each experiment was repeated at least three times. The data are presented as the mean ± SD and were analyzed by ANOVA. *P* < 0.05 was considered statistically significant.

## References

[1] Fang Q (2017). Blockade of myeloid differentiation protein 2 prevents obesity-induced inflammation and nephropathy. J. Cell Mol. Med..

[2] Kuwahara S (2016). Megalin-mediated tubuloglomerular alterations in high-fat diet-induced kidney disease. J. Am. Soc. Nephrol..

[3] Mount P (2015). Obesity-related chronic kidney disease-the role of lipid metabolism. Metabolites.

[4] Szeto HH (2016). Protection of mitochondria prevents high-fat diet-induced glomerulopathy and proximal tubular injury. Kidney Int..

[5] Ritz E, Koleganova N, Piecha G (2011). Is there an obesity-metabolic syndrome related glomerulopathy?. Curr. Opin. Nephrol. Hypertens..

[6] Yang P (2017). Inflammatory stress promotes the development of obesity-related chronic kidney disease via CD36 in mice. J. Lipid Res..

[7] Kamanna VS, Bassa BV, Ganji SH (2008). Low density lipoproteins transactivate EGF receptor: role in mesangial cell proliferation. Life Sci..

[8] Dentelli P (2007). Oxidative stress-mediated mesangial cell proliferation requires RAC-1/reactive oxygen species production and beta4 integrin expression. J. Biol. Chem..

[9] Xu T, Sheng Z, Yao L (2017). Obesity-related glomerulopathy: pathogenesis, pathologic, clinical characteristics and treatment. Front. Med..

[10] Roh DD, Kamanna VS, Kirschenbaum MA (1998). Oxidative modification of low-density lipoprotein enhances mesangial cell protein synthesis and gene expression of extracellular matrix proteins. Am. J. Nephrol..

[11] Chen HC, Guh JY, Shin SJ, Lai YH (2002). Pravastatin suppress superoxide and fibronectin production of glomerular mesangial cells induced by oxidized-LDL and high glucose. Atherosclerosis.

[12] Shen P (2017). Wedelolactone from Eclipta alba inhibits lipopolysaccharide-enhanced cell proliferation of human renal mesangial cells via NF-kappaB signaling pathway. Am. J. Transl. Res..

[13] He Y, Hara H, Nunez G (2016). Mechanism and regulation of NLRP3 inflammasome activation. Trends Biochem. Sci..

[14] Yang Qiuli, Liu Ruichen, Yu Qing, Bi Yujing, Liu Guangwei (2019). Metabolic regulation of inflammasomes in inflammation. Immunology.

[15] Yang Y (2019). Recent advances in the mechanisms of NLRP3 inflammasome activation and its inhibitors. Cell Death Dis..

[16] Wang S (2017). Salidroside alleviates high glucose-induced oxidative stress and extracellular matrix accumulation in rat glomerular mesangial cells by the TXNIP-NLRP3 inflammasome pathway. Chem. Biol. Interact..

[17] Chen MF (2018). Gigantol has protective effects against high glucose-evoked nephrotoxicity in mouse glomerulus mesangial cells by suppressing ROS/MAPK/NF-kappaB signaling pathways. Molecules.

[18] Venkatesha, S. H., Dudics, S., Astry, B. & Moudgil, K. D. Control of autoimmune inflammation by celastrol, a natural triterpenoid. *Pathog. Dis*. **74**, 1–12 (2016). 10.1093/femspd/ftw059 PMC5985506 27405485

[19] Cascao R, Fonseca JE, Moita LF (2017). Celastrol: a spectrum of treatment opportunities in chronic diseases. Front Med (Lausanne)..

[20] Ng SW (2019). Molecular modulators of celastrol as the keystones for its diverse pharmacological activities. Biomed. Pharmacother..

[21] Paimela T (2011). Celastrol regulates innate immunity response via NF-kappaB and Hsp70 in human retinal pigment epithelial cells. Pharm. Res..

[22] Hu M (2017). Celastrol-induced Nur77 interaction with TRAF2 alleviates inflammation by promoting mitochondrial ubiquitination and autophagy. Mol. Cell..

[23] Gao Yanfeng, Zhou Shuang, Pang Lizhi, Yang Juechen, Li Han John, Huo Xiongwei, Qian Steven Y. (2019). Celastrol suppresses nitric oxide synthases and the angiogenesis pathway in colorectal cancer. Free Radical Research.

[24] Xin W, Wang Q, Zhang D, Wang C (2017). A new mechanism of inhibition of IL-1beta secretion by celastrol through the NLRP3 inflammasome pathway. Eur. J. Pharmacol..

[25] Sang X (2018). Celastrol specifically inhibits the activation of NLRP3 inflammasome. Sci. China Life Sci..

[26] Guo L (2017). Targeted delivery of celastrol to mesangial cells is effective against mesangioproliferative glomerulonephritis. Nat. Commun..

[27] Zhang T (2019). Modulation of lipid metabolism by celastrol. J. Proteome Res..

[28] Zhang Y (2017). Celastrol ameliorates liver metabolic damage caused by a high-fat diet through Sirt1. Mol. Metab..

[29] Ma X (2015). Celastrol protects against obesity and metabolic dysfunction through activation of a HSF1-PGC1alpha transcriptional axis. Cell Metab..

[30] Feng X (2019). IL1R1 is required for celastrol’s leptin-sensitization and antiobesity effects. Nat. Med..

